# More Human, More Humane: A New Approach for Testing Airborne Pollutants

**DOI:** 10.1289/ehp.115-a148

**Published:** 2007-03

**Authors:** Carol Potera

## Abstract

People not only inhale airborne contaminants but also absorb them through the skin. Both routes can set off localized toxic reactions or damage internal organs such as the liver, kidney, and brain. Conventional tests of the toxicity of gases and vapors, in which laboratory animals are exposed to lethal or sub-lethal doses of chemicals, have been criticized as expensive, unethical, inhumane, and time-consuming. Now researchers at the University of New South Wales (UNSW) in Sydney, Australia, have developed an animal-free alternative that uses human cells to test the effects of exposure to airborne toxicants.

The *in vitro* method “opens new possibilities for toxicity testing of industrial chemicals, occupational and environmental contaminants, and fire combustion products,” says team leader Amanda Hayes, manager of the Chemical Safety and Applied Toxicology Laboratories in the UNSW School of Safety Science. In addition, the method could help researchers explore the health effects of nanoparticles, which increasingly are widely incorporated into cosmetics and pharmaceuticals even though “very little is known about their safety to human health,” Hayes says. This project earned Hayes and colleagues Shahnaz Bakand and Chris Winder a 2006 Australian Museum Eureka Prize, which acknowledges outstanding achievements in Australian science.

## A Cell-Based Model

In traditional *in vitro* testing, cells are grown in the bottom of a laboratory dish and covered with cell medium. Test contaminants are dissolved in the liquid medium that bathes the cells. However, this is a poor model for estimating damage from direct contact with airborne pollutants.

Hayes and coworkers improved on this method by growing human cells on Snapwell™ brand permeable polyester membranes. The cell types used, including A549 lung cells, HepG2 liver cells, and skin fibroblasts, represent target organs that are likely to be affected by airborne toxicants. Once the cells attach to the membrane and begin to flourish, the upper layer of culture medium is drawn off to expose the cells directly to air contaminants at the air–liquid interface. Meanwhile, nutrients are fed from below to keep the cells healthy.

Next the cells are exposed to airborne pollutants in a diffusion chamber. Then routine laboratory tests measure changes in cell growth and energy metabolism, along with other end points. The researchers have found that toxic measurements obtained by their in vitro method, such as the amount of a chemical needed to inhibit cell growth, mirror lethal values reported from animal studies. “In vitro toxicity tests can improve the scientific, economic, and ethical value of research and play a significant role in the replacement of animals,” Hayes says.

## Testing the Concept

In a series of experiments, the Australian team demonstrated the feasibility of their in vitro technique by testing formaldehyde, an industrial chemical linked to human cancer; nitrogen dioxide, a lung irritant that causes inflammation, pulmonary edema, and pneumonia; fire combustion products including cyanide, hydrogen sulfide, and ammonia; and xylene and toluene, two volatile organic compounds (VOCs) found in solvents used by the printing, painting, and petrochemical industries. Environmental or occupational exposure to any of these chemicals causes local and systemic toxicity.

In the VOC study, all three cell types were treated with vapors from 0, 2.5, 5, 10, 15, 20, or 30 mL of xylene or toluene for one hour. Following exposure, cell cytotoxicity was measured with the MTS assay (which measures the number of viable cells) and the NRU assay (which measures cell membrane stability). In all three cell types, airborne toluene and xylene inhibited cell growth in a dose-dependent manner, and both the MTS and NRU tests yielded similar results.

Using these results, the researchers calculated airborne IC_50_ values, or the concentration of a chemical that blocks growth of half the cells. Xylene’s IC_50_ values ranged from 5,350 to 8,200 ppm in the three cell types, making it roughly twice as toxic as toluene, with IC_50_ values of 10,500 to 16,600 ppm. These *in vitro* values correspond well to published acute inhalation data for animals. LC_50_ values (the concentration of a chemical that will kill half a group of test animals) were obtained from the NIOSH Registry of Toxic Effects of Chemical Substances for rats exposed to xylene or toluene for four hours. LC_50_ values of 5,000 ppm for xylene and 13,000 ppm for toluene correspond with IC_50_ values in the range calculated for human lung, liver, and skin cells by Hayes and her colleagues. These results appeared in the January 2006 issue of the *Journal of Environmental Monitoring*.

In another study, described in the 1 August 2006 issue of *Toxicology Letters*, the researchers exposed A549 lung cells to nitrogen dioxide concentrations ranging from 2.5 to 10 ppm. Hayes found significant adverse effects on cells at the OSHA permissible exposure level of 5 ppm, suggesting that workplace exposure standards may need to be re-evaluated.

The researchers delivered nitrogen dioxide dynamically, meaning it was constantly exchanged during the one-hour test. “This is an important refinement that mimics actual life exposure,” says epidemiologist William Lambert of Oregon Health & Science University. He explains that people are exposed to transient high levels of nitrogen dioxide from vehicle exhaust plumes while waiting at a bus stop, for example, or in the course of cooking meals on a gas stove. In contrast, traditional assays put animals in a chamber, and a known concentration of chemical is pumped in for a set amount of time.

In a third set of experiments, described in the August 2005 issue of *Toxicology in Vitro*, the researchers exposed all three cell types to 11 airborne pollutants commonly generated by fires. Fires release a variety of gases and organic vapors during their course, and 80% of fire-related deaths result from inhalation of toxic substances whose variation over the course of the fire is poorly defined. Of the compounds tested, sulfurous acid showed the greatest toxicity in all three types of cells, whereas sodium nitrate showed the least.

Several chemicals showed organ-specific action. For example, formaldehyde was twice as toxic to liver cells as it was to lung or skin cells. This highlights the importance of using a variety of target cells when testing toxic chemicals. *In vitro* tests could track the evolution of toxic substances as a fire grows, assess the safety of building materials, and provide more accurate safety information for fire professionals, the researchers concluded.

## Future Promise

The team is still validating the method in real-life settings. According to Hayes, once *in vitro* tests have been validated, the cost for the new method will be considerably cheaper than animal experimentation and considerably faster, with assay times ranging from 4 to 24 hours. Assays are performed in 96-well plates, allowing for a number of test chemicals to be assessed at once.

Alan Goldberg, director of the Center for Alternatives to Animal Testing (CAAT) at The Johns Hopkins University, says the approach “is highly focused, has clear direction, and is good science.” The new system fits the concept of the three Rs—reduction, refinement, and replacement—which are the guiding principles for scientists striving to find alternatives to animal testing. Reduction means designing experiments to use fewer animals, refinement refers to improving protocols to minimize the suffering of test animals, and replacement calls for entirely eliminating whole-animal tests. Goldberg says the alternative method being developed by Hayes and its successors “promise to reduce and possibly eliminate animals in the testing of airborne toxicants.”

After Hayes validates the *in vitro* method, she plans to develop a portable on-site test for environmental sampling and toxicity monitoring. Indeed, says Lambert, “There is a need for *in vitro* exposure systems for studying the effects of air pollutants on cells of the respiratory tract.” This type of on-site test could have helped with rescue efforts after the September 11 attack on the World Trade Center. “The complex and unique mixture of smoke and pyrolytic products could not be simulated in the laboratory, and a device like this could provide quick assessment of toxic potential,” says Lambert.

Taking such a system onsite has merit, says JeanClare Seagrave, an associate scientist at the Lovelace Respiratory Research Institute in Albuquerque, New Mexico, as long as the *in vitro* method is thoroughly validated to get reproducible responses for known exposures and includes appropriate positive and negative control treatments.

However, there are limitations to the use of such a system; these include the lack of interactions of cultured cell with the immune system or detoxification mechanisms that occur in the body. Moreover, the air is full of biological agents such as bacteria, mold, and viruses, which may deposit on cells and culture media and proliferate. Such contamination could skew results if cells react with the microbes instead of, or in addition to, toxicants. Nonetheless, says Seagrave, “Air–liquid interface exposures are clearly more physiologically relevant for lung and skin cells than conventional submersion culture systems.”

## Figures and Tables

**Figure:**
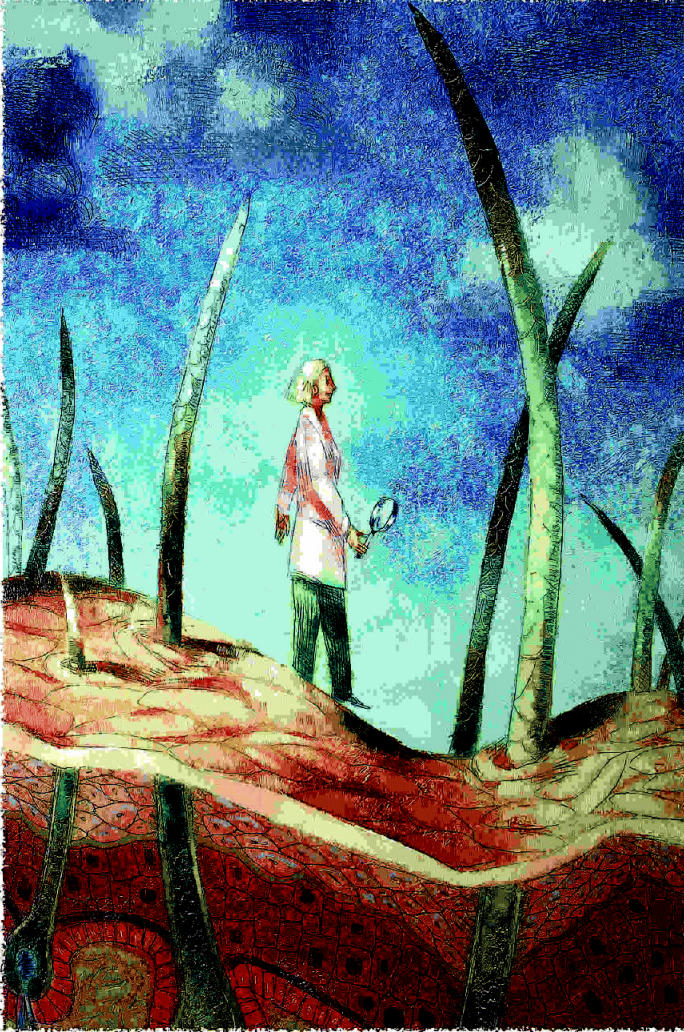


**Figure:**
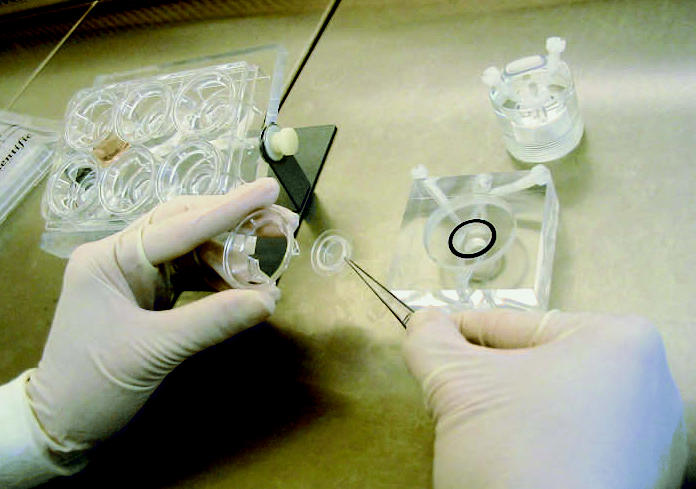
Now on the air Human cells grown on ready-made culture inserts are placed in a horizontal diffusion chamber to test the effects of airborne chemicals.
